# Executive summary of the 2020 clinical practice guidelines for the management of hypertension in the Philippines

**DOI:** 10.1111/jch.14335

**Published:** 2021-08-03

**Authors:** Deborah Ignacia D. Ona, Cecilia A. Jimeno, Gabriel V. Jasul, Ma. Lourdes E. Bunyi, Raymond Oliva, Lourdes Ella Gonzalez‐Santos, Leilani B. Mercado‐Asis, Vimar A. Luz, Aurelia G. Leus, Alejandro Bimbo F. Diaz, Marjorie I. Santos, Allan A. Belen, Dolores D. Bonzon, Jonnie Bote‐Nunez, Roberta Maria N. Cawed‐Mende, Arnel S. Chua, Anne Marie Joyce T. Javier, Dan Neftalie A. Juangco, Carmela Madrigal‐Dy, Marlon B. Manicad, Juan Miguel Gil R. Ortiz, Christia S. Padolina, Maria Concepcion C. Sison, Ninfa J. Villanueva

**Affiliations:** ^1^ University of the Philippines College of Medicine, Philippine General Hospital, Philippines; ^2^ St. Luke's Medical Center, Quezon City Philippines; ^3^ University of Santo Tomas Hospital Philippines; ^4^ Makati Medical Center Philippines; ^5^ Manila Central University‐ Filemon D. Tanchoco Medical Foundation College of Medicine Philippines; ^6^ Community General Hospital of San Pablo City Inc. Philippines; ^7^ Philippine Heart Center Philippines; ^8^ Ospital ng Paranaque Philippines; ^9^ National Kidney and Transplant Institute Philippines; ^10^ Mary Mediatrix Medical Center Lipa City Philippines; ^11^ Cardinal Santos Medical Center Philippines; ^12^ Commonwealth Hospital & Medical Center Philippines; ^13^ University of the East Ramon Magsaysay Memorial Medical Center Philippines; ^14^ Davao Medical School Foundation Philippines

**Keywords:** hypertension—general, lifestyle modification/hypertension, treatment and diagnosis/guidelines

## Abstract

Hypertension is the most common cause of death and disability worldwide with its prevalence rising in low to middle income countries. It remains to be an important cause of morbidity and mortality in the Philippines with poor BP control as one of the main causes. Different societies and groups worked and collaborated together to develop the 2020 Philippine Clinical Practice Guidelines of hypertension arising for the need to come up with a comprehensive local practice guideline for the diagnosis, treatment, and follow up of persons with hypertension. A technical working group was organized into six clusters that analyzed the 30 clinical questions commonly asked in practice, looking into the definition of hypertension, treatment thresholds, blood pressure targets, and appropriate medications to reach targets. This guideline also includes recommendations for the specific management of hypertension among individuals with uncomplicated hypertension, hypertension among those with diabetes, stroke, chronic kidney disease, as well as hypertension among pregnant women and pediatric populations. It also looked into the appropriate screening and monitoring of patients when managing hypertension, and identification of groups who are at high risk for cardiovascular (CV) events. The ADAPTE process was used in developing the statements and recommendations which were then presented to a panel of experts for discussion and approval to come up with the final statements. This guideline aims to aid Filipino healthcare professionals to provide evidence‐based care for persons with hypertension and help those with hypertension adequately control their blood pressure and reduce their CV risk

## 1 INTRODUCTION

Hypertension is a major cause of premature death worldwide and is the most important modifiable risk factor for disability adjusted life‐years lost worldwide.^1^, ^2^ The prevalence of hypertension in low and middle income countries has been seen to be steadily rising, but in the Philippines, the latest National Nutrition Survey (NNS) conducted by the Food and Nutrition Research Institute (FNRI) in 2018 showed a downward trend in hypertension prevalence for the age group 20–59 years old, from a previous of 23.9% in 2013 to 19.2% in 2018. The prevalence though for older persons aged 60 years old and above, while also decreasing, is still 35% in 2018 from 41.2% in 2015.^3^ However, hypertension awareness in the Philippines is around 67.8% and out of those who are aware, only 75% are treated with only 27% of those who are treated have it under control.^4^

Despite the decreasing trends in hypertension prevalence in the country, poor blood pressure control continues to contribute to the top two causes of mortality in the Philippines, which are heart disease and stroke. This, therefore, is the reason for the urgency of developing local practice guidelines for the management of this common disease. While there are many international practice guidelines which can be adopted in its totality, this guideline is meant to address issues that are unique and relevant to the Filipino population and when available, include local research in the development of the recommendations.

This guideline is a collaborative work of different specialties in the interest of curbing the morbidity and mortality due to hypertension in the country, by providing a set of recommendations that could guide the Filipino physician in the management of elevated blood pressure.

The objective of the guidelines is to present evidence‐based recommendations on the diagnosis and treatment of hypertension that are adapted from international practice guidelines, but which take into consideration local realities and the practice of doctors in the Philippines. It is intended to help physicians make sound clinical decisions in the management of hypertension by presenting the latest information about diagnosis, treatment, and follow‐up of persons with hypertension. The primary targets for these guidelines are physicians in general practice, but these recommendations are also useful for all healthcare professionals in the Philippines.

The guideline includes statements and recommendations on the definition of hypertension, treatment thresholds, and blood pressure targets, appropriate medications to reach targets, and specific management of hypertension among individuals with uncomplicated hypertension, hypertension among those with diabetes, stroke, chronic kidney disease, as well as the hypertension among pregnant women and pediatric populations. It also includes statements on the appropriate screening and monitoring when managing hypertension, and identification of groups who are at high risk for cardiovascular (CV) events.

## 2 METHODOLOGY

The project to develop the Philippine Practice Guideline was spearheaded by the Philippine Society of Hypertension and the Philippine Heart Association, in collaboration with experts from various fields including pediatricians and pediatric cardiologists, obstetrician‐gynecologists, endocrinologists, nephrologists, and neurologists/stroke specialists. The administrative group organized a technical writing group comprised of these experts who decided in consensus to use the ADAPTE process in developing the 2020 Clinical Practice Guidelines (CPG) on Hypertension.^5^ This decision was based not only on previous experience by other local medical societies on the use of the process for developing practice guidelines, but also because it was deemed to be an efficient, cost‐effective and evidence‐based method of making guidelines.

The rationale for guideline adaptation rather than de‐novo guideline development is to allow efficient use of current information, that is, already available in existing practice guidelines, abbreviating the process of identifying individual studies that apply to specific research questions, appraising them, evaluating individual study quality and finally, developing specific recommendations. This methodology also still allows for customizing or modifying existing guideline recommendations to suit the local context, and to add any local researches or new information on the question as they become available. The methodology for guideline adaptation using the ADAPTE process is available on the website of the Guidelines International Network at https://g‐i‐n.net . The process of adaptation is systematic, allowing for transparent and explicit reporting and typically allows the use of multiple guidelines and their contents, to develop a new set of guidelines, that is, locally relevant.

Each of the organizations involved in the process of guideline development nominated a group of experts who will comprise the technical working group (TWG). The TWG was organized into clusters that developed the six areas that were covered by the CPG: general recommendations for adults with hypertension; blood pressure management among persons with diabetes, chronic kidney disease; and stroke; and hypertension among Pregnant women and children.

### 2.1 Literature search

The TWG searched for all published guidelines, both local and international, pertaining to the clinical questions, with the use of electronic search engines and manual search. Literature search was done by each of the working groups using search engines such as Pubmed (Medline), Google Scholar, other medical search engines using key words relevant to each clinical question. The full listing of guidelines that were retrieved, appraised and included for each research question for each of the TWG clusters can be found in the full guideline.

### 2.2 Development of guideline recommendations and evidence summaries

Each cluster then developed and presented their draft recommendations for approval to the panel of experts for discussion and approval by consensus of the majority. These draft recommendations were then revised and presented again to the panel and were finalized. Several public presentations have also been made to further elicit feedback from various stakeholders on the details of the guidelines. The recommendations presented here are the result of these iterative processes.

## 3 RECOMMENDATIONS FOR THE MANAGEMENT OF HYPERTENSION

**I. Diagnosis and management of hypertension in adult population**

**Clinical question 1. Among adult Filipinos, what is the definition of hypertension?**

**Statements**:

1.1
Hypertension is defined as an office blood pressure (BP) of 140/90 mm Hg or above, typically at least twice taken on two separate days.
1.2
It is recommended that office BP be classified as Normal, Borderline, Hypertension.
1.3
Out of office BP measurements are recommended to confirm the diagnosis of hypertension, with ambulatory blood pressure monitoring (ABPM) as the preferred method, and home blood pressure monitoring (HBPM) as an acceptable alternative.

The TWG also decided to adopt the joint position statement of the Philippine Heart Association (PHA) and the Philippine Society of Hypertension (PSH)**^6^** which was a response to the 2017 ACC/AHA Guideline for the Prevention, Detection, and Management of High Blood Pressure on adults.**^7^** The 2020 Philippine Clinical Practice Guidelines (CPG) has adopted this blood pressure classification from the consensus statement as shown in Table 1.

**TABLE 1:** Blood pressure classification for adult filipinos

Category	Blood pressure range
Normal BP	< 120/80 mm Hg
Borderline BP	120–139/ 80–89 mm Hg
Hypertension	≥140/90 mm Hg

This guideline defines hypertension as an office BP of 140/90 mm Hg or above taken in accordance with the proper standard BP measurement. A cut‐off value has been set to simplify the diagnosis of hypertension and to rationalize the treatment decisions surrounding it. The continuum between BP level and the occurrence of CV and renal events is not clear, making the setting of a cut off value arbitrary. Nevertheless, hypertension is defined here as the level of BP at which the benefit of pharmacologic treatment supported by lifestyle interventions far outweigh the risks/costs of treatment as documented by clinical studies.

All the guidelines reviewed define hypertension as a BP level of ≥140/90 mm Hg, except for the AHA/ACC guideline which pegs hypertension at a BP level of ≥130/80 mm Hg. All the guidelines except for NICE and the Indian guideline include different stages and grades for hypertension. This Philippine recommendation opted to keep it simple, and the definition of hypertension remains unchanged from the 2011 Philippine guideline**^8^** because there is no compelling reason for a change. The national surveys and prevalence studies done in the Philippines use the same definition of hypertension and maintaining the same criteria would avoid confusion in disease surveillance.

**Clinical question 2. Among adult Filipinos, what device is recommended for accurate blood pressure determination and monitoring?**

**Statements**:

2.1
A properly validated automated oscillometric sphygmomanometer (digital device) is recommended for in office or out of office use.
2.2
The aneroid sphygmomanometer (manual device) may be used in office or out of office provided the examiner is efficient and well trained, and the device is periodically checked according to standard maintenance procedures.
2.3
The aneroid sphygmomanometer is recommended for special cases like the presence of arrhythmias or extremes in BP levels.

The guideline acknowledges the greater accuracy of a validated digital sphygmomanometer. However, the aneroid sphygmomanometer is still a widely available, cheaper, and accessible device for which many health care professionals have been trained. Thus, it may still be used in areas of the country where the digital device is not available, provided that there is proper training of personnel, and calibration and maintenance of the device is done regularly.

**Clinical question 3. Among adult Filipinos, what are the blood pressure thresholds for treatment and BP targets for the prevention of** CV **disease?**

**Statements**:

3.1
A therapeutic threshold of 140/90 mm Hg to achieve a goal of less than 130/80 is recommended for most adults with hypertension.
3.2
For the very elderly, defined as 80 years old and above, a therapeutic threshold of 150/90 mm Hg to achieve a goal BP of less than 140/90 mm Hg is recommended.

All guidelines agree that patients with hypertension should receive anti‐hypertensive treatment on top of diet and lifestyle modification to reduce blood pressure to treatment targets. Lowering blood pressure to less than 140/90 has been shown repeatedly to reduce morbidity and mortality.^9^, ^10^, ^11^, ^12^

This local guideline takes a practical approach to BP targets in recommending a threshold of greater than 140/90 mm Hg to start therapy. This can be addressed with diet and lifestyle modifications alone in low‐risk hypertensives or concomitant lifestyle changes with medical treatment in high‐risk individuals. **We recommend these interventions to achieve a blood pressure of < 130/80** **mm Hg in Filipino adults with hypertension**.

**Clinical question 4. Among Filipinos with hypertension, what are the general treatment recommendations?**

**Clinical question 4.1. What non‐pharmacologic approaches are recommended for persons with hypertension?**

**Statements**:

4.1
Lifestyle modification remains the cornerstone for the management of hypertension. Robust clinical trial evidence has shown that it can prevent or delay the onset of high blood pressure and can reduce CV risk. Healthy lifestyle choices are the first line of antihypertensive treatment and of course are synergistic to the effects of antihypertensive medications. Lifestyle modifications should include the following:

Sodium restriction to as low as 1500 mg/day. The American Heart Association recommends that sodium intake be limited to 2300 mg/day (about roughly half a teaspoon of table salt) in most healthy individuals and 1500 mg/day in people with prehypertension or hypertension.

4.1.1
Dietary Approaches to Stop Hypertension (DASH) meal plan which is low in sodium and high in dietary potassium, can be recommended for all patients with hypertension without renal insufficiency.^13^, ^14^, ^15^, ^16^ The DASH diet is rich in fruits, vegetables, low‐fat dairy, fish, whole grains, fiber, potassium, and other minerals at recommended levels and low in red and processed meat, sugar sweetened foods and drinks, saturated fat, cholesterol, and sodium
4.12
Aerobic physical activity and (dynamic) resistance exercises
4.13
Abstinence from alcohol or moderate alcohol intake
4.14
Significant weight loss of ≥ 5% of the baseline weight for those who are overweight or obese
4.15
Smoking cessation

Effective CV protection for hypertensive patients requires achievement of blood pressure targets with appropriate lifestyle measures and anti‐hypertensive medications. The goal of treatment strategies is to reduce excess CV morbidity and mortality from chronically elevated blood pressure.

**Clinical question 4.2. What are the preferred drugs for the treatment of hypertension among adult Filipinos for prevention of** CV **diseases?**

**Statements**:

4.2.1
Among persons with uncomplicated hypertension, angiotensin‐converting enzyme (ACE) inhibitors or angiotensin‐receptor blockers (ARBs), calcium channel blockers, thiazide/thiazide‐like diuretics are all suitable first‐line antihypertensive drugs, either as monotherapy or combination.
4.2.2
Ideal combination therapy includes renin‐angiotensin‐system (RAS) blocker with calcium channel‐blocker (CCB) or thiazide/thiazide‐like diuretics. Other combinations of the five major classes may also be used in patients with compelling indications for the use of specific drug classes.
4.2.3
ACE inhibitors & ARBs are not recommended to be used in combination. Likewise, combinations of ACE‐I or ARBs with direct renin inhibitors should not be used.
4.2.4
The use of free combinations is recommended if single‐pill combination therapy is not available or not affordable.
4.2.5
Beta blockers are suitable as initial therapy in hypertensive patients with coronary artery disease, acute coronary syndrome, high sympathetic drive and pregnant women. Beta blockers for those with congestive heart failure was specified to be bisoprolol, carvedilol, metoprolol succinate or nebivolol.
4.2.6
Among patients with BP > 150/100 mm Hg (or >160/100 mm Hg in the elderly), a combination of two agents, preferably combination of a RAAS inhibitor (ARB/ACE‐is) and CCB or diuretic, should be given initially since it is unlikely that any single agent would be sufficient to achieve the BP target.

Guideline recommendations include risk‐factor identification to stratify hypertensive patients since the presence of one or more additional CV risk factors proportionally increases the risk of coronary, cerebrovascular, and renal diseases. Risk stratification directs the degree of aggressiveness in setting BP targets and in using pharmacologic treatment on top of diet and lifestyle modifications. Risk stratification involves identification of risk factors, presence of hypertension‐mediated organ damage (HMOD) and established CV and related diseases.

Cardiovascular risk factors include advanced age (>65 years), male sex, increased body weight (BMI ≥25 kg/m^2^), diabetes, high LDL‐C (>130 mg/dl) and high triglyceride (>150 mg/dl), family history of CVD, family history of hypertension, early‐onset menopause, smoking, and various psychosocial or socioeconomic factors (poverty). **HMOD** include LVH (LVH with ECG), moderate‐severe CKD (CKD; eGFR <60 ml/min/1.73m^2^), and any other available measure of organ damage. Finally, take note of established CV and related diseases such as previous coronary heart disease (CHD), heart failure, stroke, peripheral vascular disease, atrial fibrillation, and CKD stage 3+.

The therapeutic strategy must include lifestyle changes, effective treatment of the risk factors and aggressive BP control to reach target levels to reduce the residual CV risk. Drug treatment is recommended among those with sustained systolic BP ≥140 mm Hg or diastolic BP ≥90 mm Hg despite lifestyle modification for 3 months or if HMOD is present. Patients with HMOD on screening should be started on drug treatment simultaneously with lifestyle interventions.^17^

On the basis of evidence from large‐scale clinical studies ACE inhibitors, ARBs, CCBs, and diuretics are selected as first‐line drugs.^18^, ^19^, ^20^ Overall, major CV outcomes and mortality were similar with treatment based on initial therapy with all five major classes of treatment. All five major first‐line drug classes can be combined with one another except for ACE inhibitors and ARBs. Combinations of ACE inhibitors or ARBs with either a CCB or thiazide/thiazide‐like diuretic are complementary because CCBs and diuretics activate the RAS and will also limit adverse effects associated with diuretic or CCB monotherapy. The choice for starting on initial combination therapy results in greater achievement of BP lowering at the shortest amount of time. Low‐dose combination therapy has been shown to be more effective than maximal dose monotherapy^21^

**FIGURE 1:**
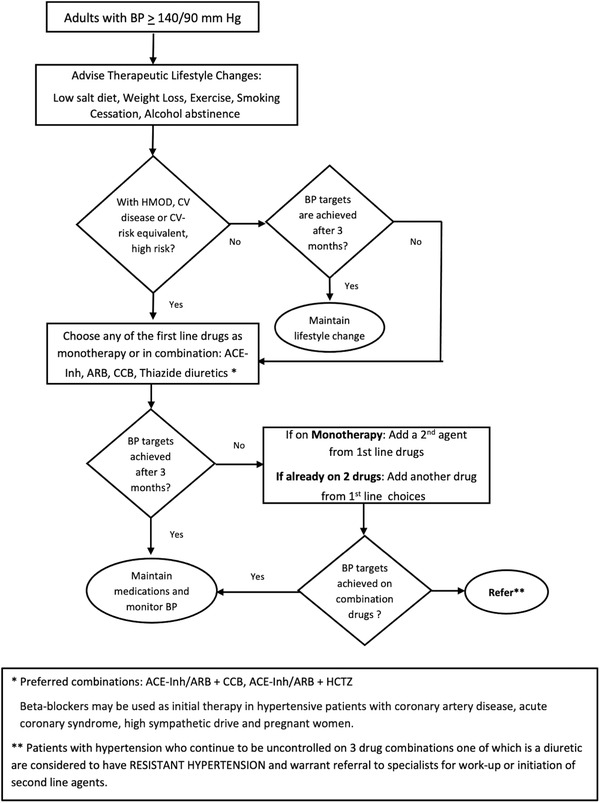
Algorithm for the management of hypertension among adults

**Clinical question** **5. Among persons with diabetes, what is the threshold for treatment of elevated blood pressure?**

**Statement**:

**5**. Among persons with diabetes and hypertension, it is recommended that drug therapy (along with lifestyle change) be initiated at a blood pressure of ≥ **140/90** **mm Hg**.

Consistent then with the section on general guidelines for treatment, plus the recommendations of majority of the international guidelines on the management of hypertension among persons with diabetes, the threshold for treatment continues to be 140/90 mm Hg.

**Clinical question 6: Among persons with diabetes and hypertension, what non‐pharmacologic therapy is recommended?**

**Statement**:

**6**. The general advice for non‐pharmacologic therapy for hypertension among persons with diabetes is similar to the general population. Additionally, screening for obstructive sleep apnea may be worthwhile as randomized studies of people with diabetes have shown that treatment of OSA (by Continuous Positive Airway Pressure or CPAP) reduces blood pressure.

**Clinical question 7. Among persons with diabetes and hypertension, what are the blood pressure targets for prevention of** CV **diseases (mortality and morbidity)?**

**Statement**:

**7**. A blood pressure target of <130/80 mm Hg is recommended for most persons with diabetes mellitus and hypertension; however, do not lower down the blood pressure below 120/70 due to an increased risk for adverse events.

While CV risk reduction (myocardial infarction, CV death) is already significant for BP <140/90 mm Hg (with no additional benefit for <120 mm Hg), a lower blood pressure target of <130/80 mm Hg has additional benefit for stroke reduction and decreased risk for nephropathy.

**Clinical question 8. Among persons with diabetes, what are the preferred drugs for the treatment of hypertension?**

**Statements**:

8.1
It is recommended to initiate treatment with a **low‐dose combination of a RAAS blocker (ACE‐I or ARB) with a CCB or thiazide/thiazide‐like diuretic**, preferably using a single‐pill combination (SPC). Free tablet combinations may also be given if SPCs are not available.

As already stated in the general guidelines, the choice for starting on initial combination therapy results in greater achievement of BP lowering at the shortest amount of time. Low‐dose combination therapy has been shown to be more effective than maximal dose monotherapy in the general population of persons with hypertension.

8.2
The combination of ACE and ARB is not recommended due to a higher risk of hyperkalemia and renal failure.

**III. Management of hypertension in persons with chronic kidney disease**

Hypertension is highly prevalent in individuals with chronic kidney disease The prevalence of hypertension increases from 36% in stage 1 to 84% in chronic kidney disease stage 4 and 5. Needless to say, blood pressure control is fundamental to the care of patients with chronic kidney disease and is relevant at all stages of chronic kidney disease regardless of underlying cause.

**Clinical question 9. Among patients with CKD who are pre‐dialysis, what is the level of blood pressure to start pharmacotherapy to prevent** CV **complications and renal progression?**

**Statement**:

**9**. Patients with BP ≥140/90 mm Hg should have prompt initiation and timely titration of pharmacotherapy to achieve blood pressure goals.

**Clinical question 10. Among patients with CKD who are pre‐dialysis, what is the target blood pressure to prevent** CV **complications and renal progression?**

**Statements**:

10.1
For *routine office blood pressure measurement*, maintain a BP target consistently <140 mm Hg systolic and <90 mm Hg diastolic in patients with low risk of CV disease and CKD grade 4 and 5, or if with adverse effect on intensive target of <130/80 mm Hg. CKD patients with high CV risk or CKD grade 3 or earlier is recommended to have a blood pressure target of <130/80 mm Hg.
10.2
A systolic BP of <120 mm Hg using a *standardized office BP measurement* is targeted, when tolerated, among adults with high BP and non‐dialysis CKD (ND‐CKD). An individualized treatment target is recommended for the following patient populations: Diabetic Kidney Disease patients, CKD grade 4 and 5ND patients, patients with proteinuria of more than 1 g/day, individuals with baseline SBP of 120 to 129 mm Hg, those with very low diastolic BP of less than 50 mm Hg with CAD, those with white coat or severe hypertension, stroke patients, those with age less than 50 with low absolute risk for CV disease or those individuals above 90 years of age, very frail patients, those with limited life expectancy and those with symptomatic postural hypotension. For these patient populations, a specialist referral is suggested.
10.3
If unable to obtain a standardized BP measure, maintain a blood pressure target consistently ≤130 mm Hg systolic and ≤80 mm Hg diastolic in patients with urine albumin excretion of >30 mg per 24 h unless adverse event occurs with achievement of this target.

**Clinical question 11. Among patients with CKD, what is the level of blood pressure to start initiation with two antihypertensive drugs to prevent** CV **complications and renal progression?**

**Statement**:

**11**. Patients with confirmed office‐based blood pressure or ≥160/100 mm Hg should, in addition to lifestyle modification, have prompt initiation and timely titration of two drugs or a single‐pill combination of drugs demonstrated to reduce CV events.

A two‐drug combination should consider these mechanisms in the choice of anti‐hypertensives: calcium channel blockers and diuretics to address volume dependent type of hypertension, and ACE, ARB and beta blockers for the renin dependent type.

**Clinical question 12. Among patients with CKD, what is the anti‐hypertensive of choice to prevent** CV **complications and renal progression?**

**Statement**:

**12**. Treatment for hypertension should include drug classes demonstrated to reduce CV events in patients with CKD such as ACE inhibitors, Angiotensin Receptor Blockers, Thiazide‐like diuretics, and dihydropyridine calcium channel blockers.

**Clinical question 13. Among patients with CKD with albuminuria/proteinuria, what is the anti‐hypertensive of choice to prevent** CV **complications and renal progression?**

**Statements**:

13.1
An ACE inhibitor or Angiotensin receptor blocker, at maximally tolerated dose is the recommended first‐line treatment for hypertension in CKD patients with urinary albumin‐to‐creatinine ratio ≥30 mg/g (or equivalent). If one class is not tolerated, the other is substituted. **These medications should not be discontinued unless serum creatinine level rise above 30% over baseline during the first 2 months of treatment or hyperkalemia (serum potassium level   ≥ 5.6** **mmol/L)**. If the patient is intolerant to both ACE inhibitor and angiotensin receptor blocker, a non‐dihydropyridine calcium channel blocker (verapamil or diltiazem) may be used as first line treatment in this setting.
13.2
Combinations of ACE inhibitor and Angiotensin receptor blocker and of ACE inhibitors or angiotensin receptor blockers with direct renin inhibitors should not be used.

**Clinical question 14. Among patients with CKD with resistant hypertension, is the addition of mineralocorticoid receptor antagonist beneficial in reducing albuminuria and** CV **events?**

**Statement**:

**14**. CKD patients with resistant hypertension not meeting blood pressure targets on three classes of anti‐hypertensive medications (including diuretic) should be considered for mineralocorticoid receptor antagonist therapy

**Clinical question 15. Among patients with CKD, is giving anti‐hypertensive at bedtime more beneficial in reducing** CV **event?**

**Statement**:

**15**. Administer one or more antihypertensive medications at bedtime

**IV. Blood pressure management among persons with stroke**

The relationship between hypertension and stroke is very dynamic and multifaceted. Management of hypertension during the acute onset of stroke (whether ischemic or hemorrhagic) and during the secondary prevention phase poses a challenge due to the intricacies of how elevated BP must be handled.

**Clinical question 16.1. For adults with acute ischemic stroke (AIS) who are eligible for intravenous (IV) thrombolysis but not for mechanical thrombectomy, what is the threshold for pharmacological treatment and the target blood pressure (BP)?**

**Statement**:

16.1
For adults with AIS who are eligible for IV thrombolysis but not for mechanical thrombectomy, a referral to a neurologist or stroke specialist is advised. It is recommended that the BP be maintained to <185/110 mm Hg prior to treatment and during infusion. For the next 24 h after treatment is given, the BP is recommended to be maintained at <180/105 mm Hg.

**Clinical question 16.2. For adults with AIS who are eligible for IV thrombolysis but not for mechanical thrombectomy, what are the pharmacologic agents of choice to reach the target BP?**

**Statement**:

16.2
It is recommended to use a titratable intravenous medication to allow dynamic adjustment of the drug depending on the current BP. For patients with acute ischemic stroke otherwise eligible for intravenous thrombolysis with BP > 185/110 mm Hg before or during infusion, or BP > 180/105 mm Hg after treatment, the recommended pharmacologic agent is Nicardipine 1–5 mg/h. IV, titrated up by 2.5 mg/h. every 5–15 min, with maximum of 15 mg/h. If available, labetalol 10 mg IV over 1–2 min followed by continuous IV infusion of 2–8 mg/min may also be used.

**Clinical question 17.1. For adults with AIS who are not eligible for IV thrombolysis or mechanical thrombectomy, what is the target BP and threshold for pharmacological treatment?**

**Statement**:

17.1
For adults with AIS who are not eligible for IV thrombolysis or mechanical thrombectomy, it is recommended to maintain a target mean arterial pressure (MAP) of 110–130 mm Hg.

For adults with AIS who are not eligible for IV thrombolysis or mechanical thrombectomy, the threshold for urgent antihypertensive treatment is with severe hypertension of Systolic BP >220 mm Hg or Diastolic BP >120 mm Hg. If with severe hypertension, it might be reasonable to reduce the BP by 15% during the first 24 h after the onset of stroke.

**Clinical question 17.2. For adults with AIS who are not eligible for IV thrombolysis or mechanical thrombectomy, what pharmacological agent may be used to achieve target BP, when needed?**

**Statement**:

17.2
For adults with AIS who are not eligible for IV thrombolysis or mechanical thrombectomy, the use of IV nicardipine to achieve the target BP may be considered.

**Clinical question 18.1. For adult patients with acute hypertensive parenchymal intracerebral hemorrhage (ICH), what is the threshold for BP lowering in the first few hours upon presentation at the emergency room?**

**Statement**:

18.1
For adult patients with acute ICH, the threshold for BP lowering is SBP ≥ 180 mm Hg.

**Clinical question 18.2. What would be the target BP when lowering the blood pressure in acute ICH?**

**Statement**:

18.2
The target SBP is <180 mm Hg. In patients with SBP ≥180 mm Hg, careful BP lowering to 140 to 160 mm Hg should be considered. The magnitude of BP reduction is dependent on the clinical context. It should be careful SBP lowering (avoiding reductions ≥60 mm Hg in 1 h).
  ■ It is recommended to keep the blood pressure stable and avoid variability.
  ■ It is also recommended not to lower the BP acutely to <140 mm Hg

**Clinical question 18.3. What are the pharmacologic agents of choice and manner of administration?**

**Statement**:

18.3
It is recommended to use intravenous antihypertensive agents that can easily be titrated to lower the BP to the desired level. The 1^st^ line drug of choice is IV Nicardipine. Alternative treatment choice would be labetalol, when available.

**Clinical question 19. For adults who have a history of stroke, what is the target blood pressure level for secondary prevention?**

**Statement**:

**19**. For adults with history of stroke, the target blood pressure level for secondary prevention is ≤ 130/80 mm Hg. RAS blockers, CCBs and thiazide diuretics remain to be the first‐line pharmacologic agents in hypertension management for secondary stroke prevention.^22^

A patient with a history of stroke will most likely have another stroke in his lifetime. In the United States, recurrent strokes make up almost 25% of the nearly 800,000 strokes annually.^23^ Hypertension remains to be the most important risk factor for both ischemic and hemorrhagic strokes. Therefore, adequate BP control plays a significant role in secondary stroke prevention.

**TABLE 2:** Summary of blood pressure thresholds and targets for persons with stroke

Context	BP threshold for initiating pharmacotherapy	Blood pressure targets	Preferred agents
**In‐hospital Mgt**	Refer to Neurologist for specialist management		Intravenous titratable anti‐hypertensives
Acute Ischemic Stroke (AIS), eligible for IV thrombolysis but not for mechanical thrombectomy	>185/110 mm Hg	<185/110 mm Hg prior to thrombolysis and during infusion; 180/105 mm Hg in the next 24 h	Nicardipine 1–5 mg/h IV, titrate up by 2.5 mg/h every 5–15 min, with maximum of 15 mg/h. If available: alternative of labetalol 10 mg IV over 1–2 min followed by continuous IV infusion of 2–8 mg/min.
AIS, not eligible for IV thrombolysis or mechanical thrombectomy	Severe hypertension: SBP of > 220 mm Hg DBP of > 120 mm Hg	If with severe hypertension, reduce the BP by 15% during the first 24 h after the onset of stroke	IV Nicardipine as indicated above
Intracerebral Hemorrhage (ICH)	SBP ≥180 mm Hg	<180 mm Hg Careful SBP lowering, avoiding reductions ≥60 mm Hg in 1 h Do not lower the BP acutely to <140 mm Hg	First choice: IV Nicardipine Second choice: IV labetalol
Secondary prevention Adults with history of stroke	140/90 mm Hg	≤ 130/80 mm Hg	First line: RAS blockers (ACE‐Inh, ARB), CCBs and thiazide diuretics

**FIGURE 2:**
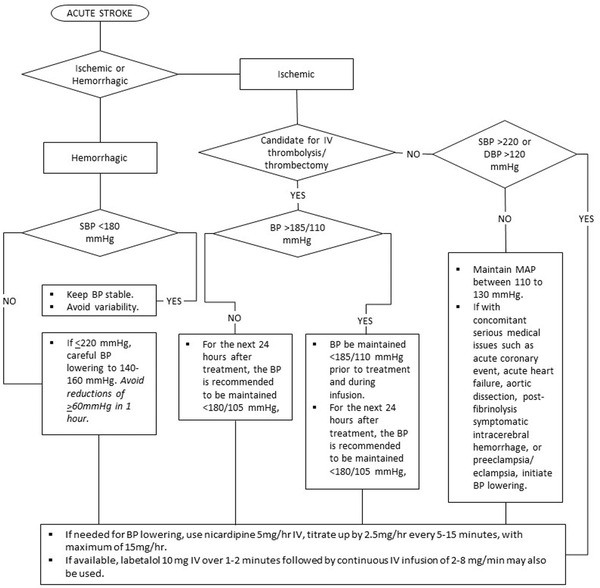
Algorithm for blood pressure management at the emergency room in acute stroke patients

**V. Management of hypertension in pregnancy**

Hypertensive disorders of pregnancy constitute one of the major causes of maternal and perinatal morbidity and mortality worldwide. It has been estimated that preeclampsia complicates 2–8% of all pregnancies globally.

**Clinical question 20. What are the different types of hypertensive disorders of pregnancy (HDP) and what are the criteria for each?** ^24^, ^25^

1. Pre‐eclampsia‐ Elevated blood pressure **and** proteinuria.
  1.1 Elevated blood pressure defined as
    1.1.1 Systolic blood pressure of 140 mm Hg or more or diastolic blood pressure of 90 mm Hg or more on two occasions at least 4 h apart after 20 weeks of gestation in a woman with a previously normal blood pressure.
    1.1.2 Systolic blood pressure of 160 mm Hg or more diastolic blood pressure of 110 m Hg or more. (Severe hypertension can be confirmed within a short interval (minutes) to facilitate timely antihypertensive therapy).
  1.2 Proteinuria (26)
    1.2.1 300 mg or more per 24 h urine collection (or this amount extrapolated from a timed collection), or
    1.2.2 Protein/creatinine ratio of 0.3 mg/dl or more or
    1.2.3 Dipstick reading of 2+ (used only if other quantitative methods not available)
  1.3 Or in the absence of proteinuria, new onset hypertension with the new onset of any of the following:
    1.3.1 Thrombocytopenia: Platelet count < 100,000 × 10^9^/L
    1.3.2 Renal insufficiency: Serum creatinine concentrations >1.1 mg/dl or a doubling of the serum creatinine concentration in the absence of other renal disease
    1.3.3 Impaired liver function: Elevated blood concentrations of liver transaminases to twice normal concentration
    1.3.4 Pulmonary edema
    1.3.5 New‐onset headache unresponsive to medication and not accounted for by alternative diagnoses or visual symptoms
  1.4 Eclampsia‐ New‐onset tonic‐clonic, focal, or multifocal seizures in the absence of other causative conditions such as epilepsy, cerebral arterial ischemia and infarction, intracranial hemorrhage, or drug use.
  1.5 Chronic Hypertension‐ Hypertension of any cause, that predates pregnancy. BP ≥ 140/90 mm Hg before pregnancy or before 20 weeks gestation or both.
  1.6 Chronic Hypertension with Superimposed Pre‐eclampsia‐ Chronic hypertension in association with preeclampsia. Others define it as worsening baseline hypertension accompanied by new‐onset proteinuria or other findings supportive of preeclampsia.

For patients with chronic hypertension, it can be difficult to differentiate worsening of the hypertension from superimposed preeclampsia. Conditions that may indicate superimposed preeclampsia, that warrants a referral to a maternal fetal medicine specialist/perinatologist, include the following:

1. Acute, severe, and persistent elevations in blood pressure.
2. Sudden increase in baseline hypertension.
3. New‐onset proteinuria or sudden increase in proteinuria.
  1.7 Gestational Hypertension –
    1.7.1 Systolic blood pressure 140 mm Hg or more or a diastolic blood pressure of 90 mm Hg or more, or both, on two occasions at least 4 h apart after 20 weeks of gestation, in a woman with a previously normal blood pressure.
    1.7.2 Hypertension without proteinuria or severe features develops after 20 weeks of gestation and blood pressure

**Clinical question 21. What blood pressure threshold is used to define hypertension in pregnancy?**

**Statement**:

**21**. Hypertension is diagnosed empirically when appropriately taken blood pressure is 140 mm Hg systolic or 90 mm Hg diastolic or above. Korotkoff phase V is used to define diastolic pressure.

**Clinical question 22. What antihypertensive agents can be used for urgent blood pressure control in pregnancy?**

**Statement**:

**22**. Acute‐onset severe hypertension (systolic BP of 160 mm Hg or more or diastolic BP of 110 mm Hg or more, or both) can occur in the prenatal, intrapartum and postpartum period. It is accurately measured using standard techniques and is persistent for 15 min or more. The first line of treatment is intravenous (IV) hydralazine and labetalol; intravenous nicardipine is also an option. Extended release oral nifedipine also may be considered as a first line therapy, particularly when IV access is not available. Use of these drugs does not require cardiac monitoring.

**Clinical question 23. When do we treat hypertension during pregnancy?**

**Statement**:

**23**. Treatment of severe hypertension (blood pressure of ≥ 160/100 mm Hg) is always recommended as it prevents serious maternal and fetal complications to set in.

Initiating therapy in non‐severe disease, however, is a patient of controversy. The NICE, ISSHP and SOGC recommends therapy when the blood pressure remains above 140/90 mm Hg but SOGC suggests a lower threshold in patients with other co‐morbidities.^26^, ^27^, ^28^, ^29^ The ACOG recommends conservative management of non‐severe disease but stressed on the importance of control in the severe type.^30^ It is also important to avoid hypotension because the degree by which placental blood flow is auto‐regulated is not established, and aggressive lowering may cause fetal distress.

**Clinical question 24. What are the pharmacologic treatment options in the OPD management of hypertension in pregnancy?**

**Statement**:

**24**. The choice of antihypertensive drug for initial therapy should be based on the characteristics of the patient, contraindications to a particular drug and physician and patient preferences. The first line drugs are methyldopa, calcium channel blockers or beta blockers, and ACE‐inhibitors and angiotensin‐receptor blockers (ARBs) are generally not recommended. Antihypertensives may be used to keep systolic blood pressure at 130 to 155 mm Hg and diastolic blood pressure at 80 to 105 mm Hg.

**Clinical question 25: How is hypertension managed during the immediate postpartum and breastfeeding periods?**

**Statement**:

**25**. Blood pressure should be recorded shortly after birth and if normal again within 6 h.

All women should have BP recorded and discharge deferred for at least 24 h or until vital signs are normal and/or treated or referred. Any woman with an obstetric complication and/or newborn with complications should stay in the hospital until both are stable.

- In hospital stay for at least 24 h
- Checkup within 48–72 h of the birth and again 7–14 days and at 6 weeks post‐partum.
- All women should be reminded of the danger signs of preeclampsia following birth including headaches, visual disturbances, nausea, vomiting, epigastric or hypochondrial pain, feeling faint or convulsions.

**VI. Blood pressure management in the pediatric population**

**Clinical question 26**. Among pediatric patients, what is the threshold for commencing pharmacologic treatment for Hypertension?

**Statements**:

**26**.1 Pharmacologic treatment for hypertension (HTN) should be started for children with the following conditions:

26.1.1 Children who remain hypertensive even after 6 months of lifestyle modification strategies* (see Table 3
26.1.2 Symptomatic hypertension or Stage 2 hypertension
26.1.3 Presence of co‐morbidities like chronic kidney disease (CKD) or diabetes mellitus (DM), or any evidence of target organ involvement (eg, left ventricular hypertrophy).

**26.2**. The goal of pharmacologic therapy should be a reduction in systolic blood pressure (SBP) and diastolic blood pressure (DBP) to <90^th^ percentile for age, sex and height and <120/80 mm Hg in adolescents ≥13 years of age.

**26.3**. For children with CKD, BP targets should be less than or equal to 50^th^ percentile for age, sex and height.

**26.4**. The goal of treatment of hypertension in the pediatric population is not only to reduce BP to <90^th^ percentile for age, sex and height and <130/80 mm Hg, but also to reduce CV risk factors, and prevent target organ damage.

**26.5**. Follow‐up every 4–6 weeks is recommended for monitoring and evaluation of therapy.

**TABLE 3:** Blood pressure stages in filipino pediatric population^31^)

Filipino children 1–13 years	Filipino children ≥ 13 years
Normal BP: <90th percentile	Normal BP: <120/<80 mm Hg
Elevated BP: ≥90th percentile to <95th percentile or 120/80 mm Hg to <95th percentile (whichever is lower)	Elevated BP: 120/<80 to 129/<80 mm Hg
Stage 1 HTN: ≥95th percentile to <95th percentile + 12 mm Hg, or 130/80 to 139/89 mm Hg (whichever is lower)	Stage 1 HTN: 130/80 to 139/89 mm Hg
Stage 2 HTN: ≥95th percentile + 12 mm Hg, or ≥140/90 mm Hg (whichever is lower)	Stage 2 HTN: ≥140/90 mm H

**Clinical question 27. What advice regarding nonpharmacologic treatment is recommended for pediatric patients?**

**Statements**:

**27.1** Non‐pharmacologic therapy of lifestyle modification which include Dietary Approaches to Stop Hypertension (DASH) and engaging in 30–60 min of moderate to vigorous physical activity at least 3–5 days a week should be initiated in all pediatric patients consulting for the first time for hypertension.

**27.2** All children with hypertension should have their body mass index (BMI) measured during each visit.

**27.3** Weight loss intervention is recommended for identified overweight and obese children until a normal BMI is attained through dietary counselling and exercise (weight loss of 1 to 2 kg per month).

**27.4** All children diagnosed to have hypertension or elevated BP should be advised to do the following:

27.4.1 Decrease intake of high sodium content and calorie‐dense food and beverages, and to increase intake of fruits and vegetable to 3–5 servings per day.
27.4.2 Engage in moderate‐to‐vigorous exercise 30–60 min at least 3–5 times a week but preferably daily, unless with medical contraindication.
27.4.3 Avoid smoking including electronic cigarettes and exposure to tobacco smoke.
27.4.4 Avoid alcohol intake and caffeinated energy drinks.

**Clinical question 28. What are the BP targets for prevention of target organ complications?**

**Statements**:

**28.1** The target BP for children is <90^th^ percentile for age, sex and height or <120/<80 mm Hg whichever is lower.

**28.2** For CKD patients, BP target is less than or equal to the 50^th^ percentile for age, sex and height.

**Clinical question 29. What are the preferred medications for children?**

**Statements**:

**29.1** Any of the following drugs may be used as initial treatment for children with hypertension: ACE inhibitors (Enalapril, Captopril), ARBs (Losartan, Valsartan), or calcium channel blockers (Amlodipine).

**29.2** For children with co‐existing chronic kidney disease, proteinuria or diabetes mellitus, an ACE‐inhibitor or ARB is recommended as the initial antihypertensive drug unless with absolute contraindications. Referral to a specialist is highly recommended.

**29.3** Therapy should start with a single drug at the lowest possible dose and titrated up every 2–4 weeks until target BP is achieved, or the maximal dose reached, or adverse effects occur.

**29.4** If BP is not controlled with a single agent (maximal dose is reached or adverse effects occur), a second agent can be added to the regimen and titrated as with the initial drug. Because the use of other anti‐hypertensive agents can lead to compensatory salt and water retention, the addition of a thiazide diuretic to an initial drug for uncontrolled hypertension is prudent.

**29.5** In combining agents from different drug classes, it is preferable to give those with complementary modes of action. Ideally, no two drugs which act separately on the RAAS, should be used in combination because of the risk of hyperkalemia, impaired kidney function and hypotension.

**Clinical question 30: What is the recommended technique and BP device for accurate BP measurement in pediatric patients?**

**Statements**:

**30.1** The use of proper technique and appropriately‐sized cuff is critical for the accurate measurement of BP in children.

**30.2** An auscultatory device using an aneroid non‐mercury sphygmomanometer is recommended for children.

**30.3** An oscillometric device is a suitable alternative to auscultation for initial BP screening and monitoring in the pediatric population.

**30.4** Ambulatory BP monitoring (ABPM) is recommended in children (> 5 years old) and adolescents with the following conditions:

30.4.1 Elevated office BP measurements for one or more years, or if with stage 1 hypertension over 3 clinic visits, for confirmation of hypertension.
30.4.2 Those with high‐risk conditions (eg, obesity, CKD or structural renal abnormalities, diabetes mellitus, those who have undergone solid organ transplant, obstructive sleep apnea, repaired aortic coarctation) to document masked hypertension.
30.4.3 Those with *suspected* white coat hypertension (WCH).

**30.5** Home BP monitoring should not be used to diagnose hypertension, MH, or WCH but may be a useful adjunct to office and ambulatory BP measurement if clinically validated oscillometric apparatus and appropriate‐sized cuffs are used.

**FIGURE 3:**
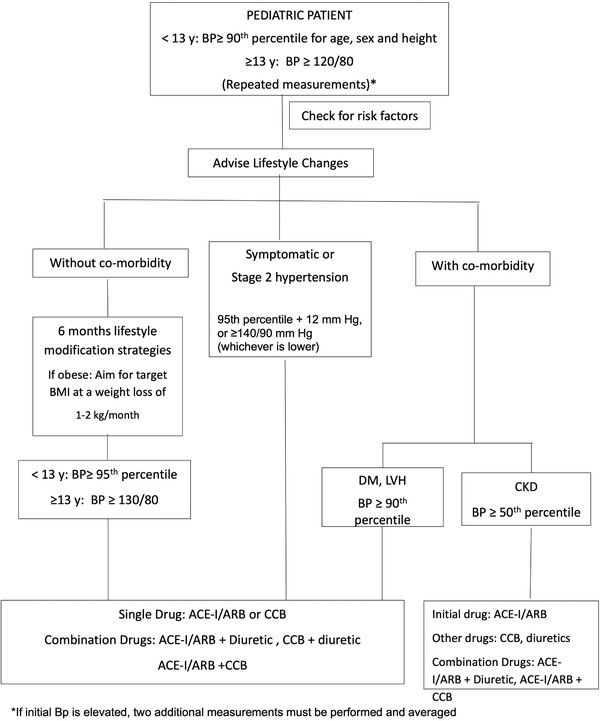
Treatment algorithm for hypertension in the pediatric population

## 4 CONCLUSIONS

The clinical questions and statements in this clinical practice guideline allow us to holistically manage the Filipino hypertensive individual. We recommend that the diagnosis of hypertension among adults should be based on a BP reading of equal or more than 140/90 mm Hg. The target BP should be less than 130/80 mm Hg to prevent hypertension‐mediated organ damage. We also recommend that lifestyle modification be advised to all persons with hypertension. The use of appropriate anti‐hypertensive medications depending on the co‐morbidity of the individual is recommended. We also recommended management for the pediatric and the pregnant hypertensive. Simplified algorithms are provided to serve as a quick reference guide to clinicians. There was limited Filipino data, particularly in the pediatric population thus, local studies are needed to collect real‐world data on the use of various medications in various populations, and even of the utility of this practice guideline.

The 2020 CPG is designed to be a guide for clinicians in managing hypertension for the Filipino patient. This, however, should not replace sound clinical judgment, and the ultimate decision for treatment should be shared by both clinician and the patient.

## STEERING COMMITTEE

Dr. Gilbert C. Vilela (Philippine Heart Association)

Dr. Alberto A. Atilano (Philippine Society of Hypertension)

Dr. Aurelia G. Leus (Philippine Heart Association)

Dr. Felix Eduardo R. Punzalan (Philippine Society of Hypertension)

**Expert Panel**

(Cardiology Group)

Joanna Cosare‐San Pedro, MD

Julius Caesar D. De Vera, MD

Ramanaya D. Garcia, MD

Joanna Teresa Margarita L. Manalo, MD

Irma Marie P. Yape, MD

Christian Michael H. Pawhay, MD

Eduardo O. Yambao Jr., MD

(Nephrology Group)

Christine V. Pascual, MD

Marichel Pile‐Coronel, MD

Gelen Vestalez Umali‐Sunga, MD

(Pediatrics Group)

Emely G. Anupol, MD

Juliet J. Balderas, MD

Jose Jonas D. Del Rosario, MD

Bee Jane T. Martinez, MD

Leah Patricia A. Plucena, MD

Andrea Orel S. Valle, MD

Eleanor C. Du, MD (Philippine Society of Pediatric Metabolism and Endocrinology)

Sylvia C. Estrada, MD (Philippine Society of Pediatric Metabolism and Endocrinology)

Maria Rosario F. Cabansag, MD (Pediatric Nephrology Society of the Philippines )

Lorna Lourdes L. Simangan, MD (Pediatric Nephrology Society of the Philippines )

Dorothy M. Ann Calma, MD (Child Neurology Society, Philippines)

(Obstetrics Gynecology Group)

Joseph U. Olivar, M.D.

Kristine S. Sese, M.D.

Irene B. Quinio, M.D.

Ma. Geraldine Assumption Gavina C. Torralba, M.D.

Zarinah G. Gonzaga, M.D.

Amaryllis Digna O. Yazon, M.D.

Nerissa Gracia G. Nano‐de Guzman, M.D.

## FINANCIAL DISCLOSURES

Drs. Leilanie Mercado‐Asis, Vimar Luz, Aurelia Leus, Alejandro Bimbo Diaz, Allan Belen, Roberta Maria Cawed‐Mende, Arnel Chua, Anne Marie Joyce Javier, Dan Neftalie Juangco, Juan Miguel Ortiz, Christia Padolina, Maria Concepcion Sison, Jonnie Bote‐Nunez, Carmela Madrigal‐Dy, Marlon Manicad,Ninfa Villanueva have nothing to disclose.

Dr. Deborah Ignacia Ona receives honoraria as part of the speaker's bureau of Upjohn, Viatris, Astra‐Zeneca, Sanofi, Servier, MSD, Getz, Advisory Board of Sanofi, research grant from St. Luke's Medical Center, outside the submitted work

Dr. Cecilia Jimeno is the vice editor of JAFES; reports speaking engagements of Natrapharm, Novartis, Servier Philippines, LRI‐Therapharma; CME grant from OEP, Cathay; Advisory board of MSD, outside the submitted work.

Dr Gabriel Jasul reports honorari as part of the speaker's bureau of Boehringer Ingelheim , MSD, Sanofi‐Aventis, Novo Nordisk, LRI‐Therapharma, Abbott Nutrition, Umed, Astra Zeneca, and J &J

Dr. Ma. Lourdes Bunyi receives speaking grants from Omron.

Dr. Raymond Oliva reports connection to Astellas Pharma Philippines, outside the submitted work.

Dr. Lourdes Ella Gonzalez‐Santos reports personal fees for speaking engagements from LRI‐Therapharma, Sanofi‐Aventis, Servier Philippines, Merck Sharpe and Dohme, Astra Zeneca, Menarini, Novartis, Natrapharm, Pascual, Amgen; research grant from Cardinal Santos Medical Center, outside the submitted work.

Dr. Dolores Bonzon is receiving speaking grants from Viatris.

All authors of this manuscript fulfilled the ICMJR criteria for authorship:

- Substantial contributions to the conception or design; or the acquisition, analysis or interpretation of data for the work
- Drafting the work or revising it critically for important intellectual content
- Final approval of the version to be published
- Agreement to be accountable for all aspects of the work in ensuring that questions related to the accuracy or integrity of any part of the work are properly investigated and resolved
